# Supplementary material for: Factors affecting repurchase intention of organic food among generation Z (Evidence from developing economy)
Source: PLoS One. 2023 Mar 24;18(3):e0281527. doi: 10.1371/journal.pone.0281527 (PMC10038270; doi:10.1371/journal.pone.0281527)
Supplement: S1 Appendix — (DOCX) [file pone.0281527.s001.docx]

APPENDIX

|  | Brand purchase |  |  |  |  |  |
| --- | --- | --- | --- | --- | --- | --- |
| 1 | I intend to buy green products | 1 | 2 | 3 | 4 | 5 |
| 2 | I plan to purchase green products | 1 | 2 | 3 | 4 | 5 |
| 3 | I will purchase green products in my next purchase | 1 | 2 | 3 | 4 | 5 |
|  | Social media marketing |  |  |  |  |  |
| 1 | The social media advertisements for this green's brand are frequently seen. | 1 | 2 | 3 | 4 | 5 |
| 2 | The level of the social media advertisements for this green brands meets myexpectations. | 1 | 2 | 3 | 4 | 5 |
| 3 | The social media advertisements for this green brand are very attractive. | 1 | 2 | 3 | 4 | 5 |
| 4 | The social media advertisements for this green brand perform well in comparison to those of other restaurants. | 1 | 2 | 3 | 4 | 5 |
| 5 | This green's brand offer extensive advertisement on social media. | 1 | 2 | 3 | 4 | 5 |
| 6 | The social media advertisements for the brand of this green brand can be easily | 1 | 2 | 3 | 4 | 5 |
|  | Green brand awareness |  |  |  |  |  |
| 1 | You can recognize green brands among other competing brands because of their environmental commitments | 1 | 2 | 3 | 4 | 5 |
| 2 | You are aware of green brands because of environmental reputation | 1 | 2 | 3 | 4 | 5 |
| 3 | Some of environmental characteristics of green brands come to top of mind in your consideration set quickly | 1 | 2 | 3 | 4 | 5 |
| 4 | You can quickly recall the image of green brands | 1 | 2 | 3 | 4 | 5 |
| 5 | You can quickly figure out Green brand because of environmental concern | 1 | 2 | 3 | 4 | 5 |
|  | Consumer satisfaction |  |  |  |  |  |
| 1 | The staying experience at that hotel made me satisfied. | 1 | 2 | 3 | 4 | 5 |
| 2 | My choice to stay at that hotel was a wise one. | 1 | 2 | 3 | 4 | 5 |
| 3 | Overall, I feel satisfied about that hotel. | 1 | 2 | 3 | 4 | 5 |
|  | Repurchase intention |  |  |  |  |  |
| 1 | I anticipate to repurchasing in near future | 1 | 2 | 3 | 4 | 5 |
| 2 | It is likely that I will repurchase in near future | 1 | 2 | 3 | 4 | 5 |
| 3 | I expect to repurchase in the near future | 1 | 2 | 3 | 4 | 5 |
