# Supplementary material for: Factors affecting repurchase intention of organic food among generation Z (Evidence from developing economy)
Source: PLoS One. 2023 Mar 24;18(3):e0281527. doi: 10.1371/journal.pone.0281527 (PMC10038270; doi:10.1371/journal.pone.0281527)
Supplement: Attachment — Submitted filename: changes (1).docx [file pone.0281527.s002.docx]

| Serial No. | Changing suggested | Incorporated at |
| --- | --- | --- |
|  | Reviewer One |  |
|  | Required citation | Page no. 5 Fifth paragraph, line no. 198-99;  page no. 5second paragraph, line no. 170-71;  page no. 5second paragraph, line no. 171-72;  page no. 1 second paragraph, line no38-39 and  pageno.2 third paragraph, line no. 77-78 |
|  | Refereeing generation Z theory | Page no. 2 third paragraph line no. 78 |
|  | Money in USD | page no. 6fourth paragraph, line no. 232-34 |
|  |  |  |
|  |  |  |
|  | Reviewer two |  |
|  | APA formatting | References are updated accordingly |
|  | Manuscript proof reading | Incorporated |
|  | Biswas & Roy[3] | Changed according to APA formatting |
|  | Write all statistics in one paragraph | Page two paragraph no.2 |
|  | Page 2 line 50,cite properly | Follow APA style |
|  | Generation Z | page no. 9 paragraph no. 5, line no. 307 |
|  | Add reference | page no. 2 third paragraph, line no. 78 |
|  | Customer satisfaction | Page no. 5 Fourth paragraph, line no. 195;  page no. 5 second paragraph, line no. 201-02 |
|  | Brand awareness | Page no. 5 third paragraph, line no. 183-84;  page no. 5 third paragraph, line no. 187 |
|  |  |  |
|  | Practical contribution | page no. 10first paragraph, line no. 320-23; |
|  | Future recommendation | Page no. 10 paragraph no.3 line no. 344-48 |
|  | Bench mark value | Page no. 3 paragraph no.2 line no. 96-97 |
|  |  |  |
|  |  |  |
|  |  |  |
|  |  |  |
|  |  |  |
|  |  |  |
|  |  |  |
|  |  |  |
|  |  |  |
|  |  |  |
|  |  |  |
